# Current updates on adaptive immune response by B cell and T cell stimulation and therapeutic strategies for novel coronavirus disease 2019 (COVID-19) treatment

**DOI:** 10.1016/j.heliyon.2021.e06894

**Published:** 2021-04-27

**Authors:** Neeraj Pal, Anil Kumar Mavi, Sundip Kumar, Umesh Kumar, Maya Datt Joshi, Rohit Saluja

**Affiliations:** a Department of Molecular Biology and Genetic Engineering, GB Pant University of Agriculture and Technology, Pantnagar, 263145, India; b Department of Pulmonary Medicine, Vallabhbhai Patel Chest Institute, University of Delhi, 110007, India; c School of Biosciences, IMS Ghaziabad University Courses Campus, Uttar Pradesh, 201015, India; d Department of Biotechnology, Shobhit Institute of Engineering & Technology (Deemed to be University), Meerut, 250110, India; e Department of Biochemistry, All India Institute of Medical Sciences, Bibinagar, Hyderabad, Telangana, 508126, India

**Keywords:** COVID-19, B cell, T cell, Vaccine, Drugs

## Abstract

The prevalence of COVID-19 continues to rise with more than 114,315,846 million confirmed cases and 2,539,427 deaths worldwide by 3 March 2021 and this number kept on increasing day by day. There is no clear therapeutic treatment or vaccine available for COVID-19 till date and by seeing such a high rise in the cases of COVID-19 on daily basis, it would have been necessary to implement precautions and hygienic measures to monitor and reduce human-to-human transmission of SARS-CoV-2 before there is any successful intervention/treatment available. Currently, several studies demonstrated the important improvements in both the innate and adaptive immune systems of COVID-19 patients. In particular, pre-existing research, on immune response to B cell and T cells are highlighting that pre-existing immunity exists in about 90% of the general population because of previous exposure to CoVs and having immunity against these CoVs. Although it is not clear from, the current studies on COVID-19 but it assumed that, it might have implication to COVID-19 severity and could play an important role in treatment or vaccine development against COVID-19. This review summarizes the information from occurrence of SARS-CoV-2 to its pathogenesis, transmission, adaptive immune response with respect to T cell and B cell stimulation and therapeutic interventions/treatment against COVID-19.

## 1 Introduction

Coronaviruses were first identified in the 1930s when there was evidence of an acute respiratory infection in domesticated newborn chickens caused by Infectious Bronchitis Virus (IBV) (Estola, 1970). In 1931, Arthur Schalk and M.C. Hawn described these newborn chickens respiratory infection in North Dakota and characterized it by gasping and listlessness with chicks' mortality rate of 40–90% (Fabricant, 1998). Later, Fred Beaudette and Charles Hudson successfully isolated and cultivated the infectious bronchitis virus after six years (Decaro, 2011). Two more animal CoVs were isolated in the 1940s after that, the mouse hepatitis virus (MHV) and the transmissible gastroenteritis virus (TGEV) (McIntosh, 1974). At the time, it was not known that these three distinct viruses were related. In 1965, the history of human CoV began when Tyrrell and Bynoe discovered that they could passage a virus called B814 contained in human tracheal organ cultures obtained from an adult with the common cold's respiratory tract. While working on B814 and human virus type 229E infectivity, it was found that these are ether-sensitive and require lipid-containing coat for infection, but none of the viruses were associated with any known myxo or paramyxovirus. In 1967, McIntosh et al., reported the recovery from human respiratory tract of several strains of ether-sensitive agents, and called them "OC '' to indicate they were grown in organ culture. At the same time as electron microscopy was performed on fluid from organ cultures contaminated with B814, some particles are detected that resembles IBV, MHV and TGEV, all of which proved morphologically similar and identified as the new group of viruses called human CoV (Kahn and McIntosh, 2005; Bradburne, 1969).

Corona viruses are the member of the family Coronaviridae. They infect both humans and diverse animals, including birds and mammals (Channappanavar and Perlman, 2017). There have been seven human coronaviruses (HCoV) identified to date. Including HCoV-NL63 and HCoV-229E alpha-CoV and HCoV-OC43 beta-CoV, HCoV-HKU1, extreme acute respiratory syndrome (SARS-CoV), Middle East respiratory syndrome-CoV (MERS-CoV), and newly emerged SARS-CoV-2 (Yin and Wunderlink, 2018). Three of these triggered epidemic diseases with rapid spread leading to global health emergency worldwide: the Severe Acute Respiratory Syndrome Coronavirus (SARS-CoV) emerged in 2002 in the province of Guangdong, China, which was subsequently associated with 8,096 cases and 774 deaths; the Middle East Respiratory Syndrome Coronavirus (MERS-CoV) emerged in 2012 in Saudi Arabia with the global spread of 2,494 cases and 858 deaths. and now the new emerging COVID-19 (SARS-CoV-2) in 2019 in Wuhan city, Hubei province, China with the global spread of 114,315,846 confirmed cases, including 2, 539,427 deaths by 3 March 2021 among 203 countries or regions with confirmed cases-50,824,766 (Americas), 38,931,803 (Europe), 13,581,219 (South-East Asia), 6,482, 557 (Eastern Mediterranean), 2,857,860 (Africa), 1,636,896 (Western Pacific) and the number is still kept on increasing on daily basis (Hoffmann et al., 2020; Wit et al., 2016; https://covid19.who.int/). For example, in the case of SARS-CoV-2, SARS-CoV-2 has spread worldwide following the outbreak in China. As of early April 2020, the number of COVID-19 patients registered is highest in the United States, followed by Spain, Italy, Germany, France and China, and now in India. After China's outbreak, Italy was greatly affected (Onder et al., 2020). The fatality rate was initially higher in the elderly population and the percentage of children among the total COVID-19 patients was small. Later the infection was reported with a high rate in every age group. According to the Chinese Disease Control and Prevention Center (CDC) report, 2134 pediatric patients with COVID-19, 4.4%, 50.9%, 38.8% and 5.9% of patients were diagnosed as asymptomatic, mild, moderate or severe during infection, respectively. Table 1 below summarizes the concept of asymptomatic, mild, moderate, extreme and critical.

**Table 1:** Classification of Covid-19 patients.

Asymptomatic	Without any clinical symptoms, but may be shedding substantial amounts of virus.
Mild	Symptoms of acute infection of the upper respiratory tract (fever, fatigue, myglia, cough, sore throat, runny nose and sneezing) or gastrointestinal symptoms (nausea, vomiting, stomach pain, diarrhoea).
Moderate	Pneumonia (frequent fever, cough) and no noticeable hypoxia.
Severe	Pneumonia with hypoxia (Spo2 < 92 per cent)
Critical	There may be shocks i.e., Acute respiratory distress syndrome (ARDS), encephalopathy, heart failure/myocardial injury, coagulation dysfunction, and acute kidney injury.

High-rise in COVID-19 cases has been reporting on a regular basis, as we are looking at today. The COVID-19 therapies are frequently ineffective, and there are currently no appropriate vaccines or treatment to prevent the disease. As it is said that, clarification of immune responses is key to the creation of strategies for diagnosis, regulation, and prevention. Furthermore, studies of humoral and cellular immunities may lead to the identification of the antigens and epitopes responsible for inducing protective immune responses against SARS-CoV-2. Such knowledge is essential for successful vaccine development and vaccination strategies. Reports of worldwide immune responses following SARS-CoV-2 exposure are minimal. Researchers have been exploring around the results of the Common Cold Study to understand the human immune response to SARS-CoV-2 along with the latest molecular techniques with the use of animals and the cell culture (Ledford, 2020). Moreover, it is not clear what role host immunity plays against SARS-CoV-2 in viral clearance or tissue damage. High initial viral load has been shown to be independently associated with disease severity and can be influenced by immune responses from the host. Recent studies have, however indicated that SARS-CoV-2 B cell and T cell may play a key role in switching from innate immunity to adaptive immunity during an acute phase. Here in this review we have listed few of the current and pre-existing studies, showing that looking into the immune response to B cells and T cells could be one of the ways to provide defense against COVID-19 together with current updates on vaccines and repurposing drugs under clinical evaluation.

## 2 Genome organization and genome characterization of SARS-CoV-2

**Figure 1:**
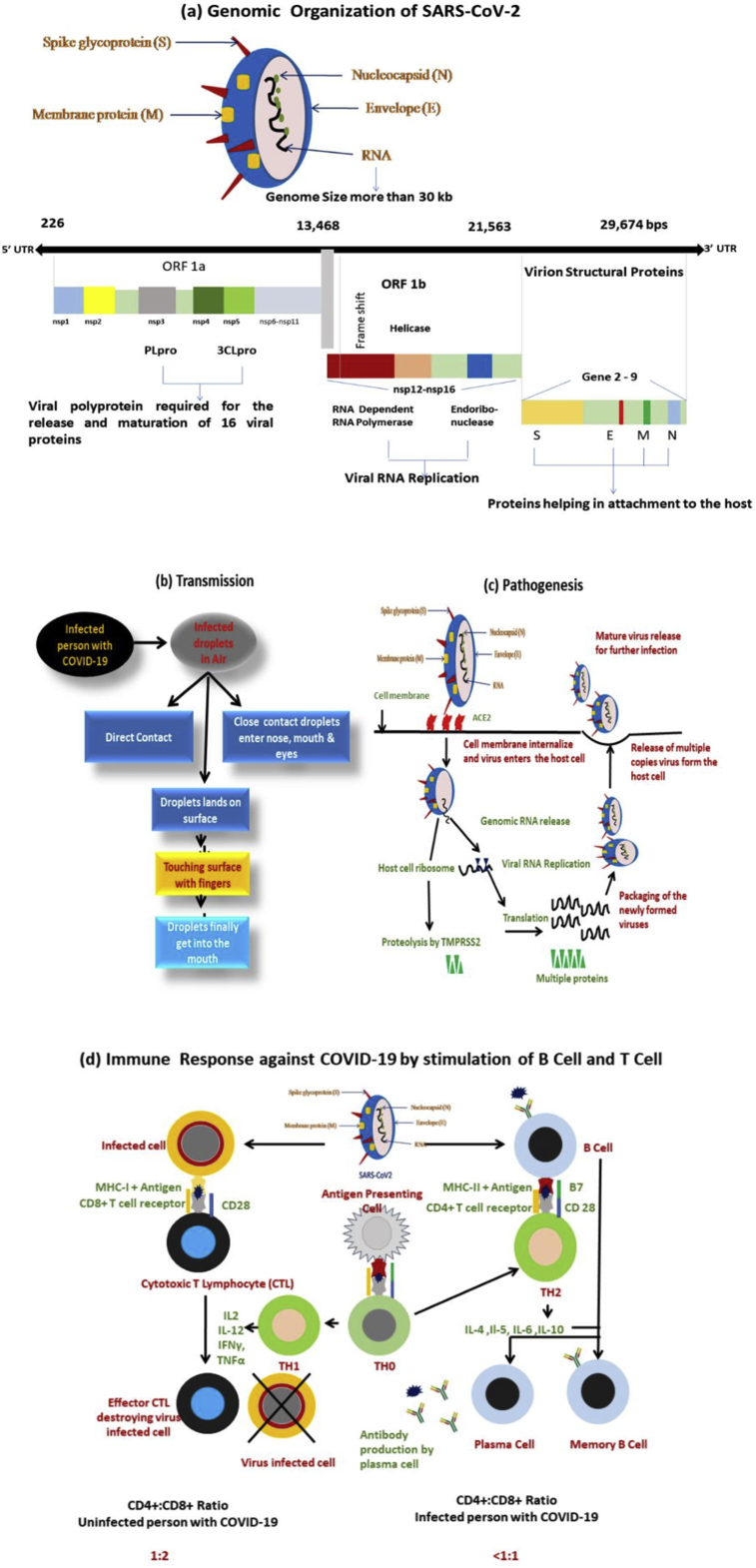
(a) Structure of coronavirus showing spike protein (S), membrane protein (M), envelope protein (E), lipid bilayer, nucleocaspid (N), RNA and Genomic organization of the SARS CoV-2. (b) Mechanism of transmission of SARS CoV-2 infection. (c) Pathogenesis of SARS CoV-2 in Host cell. (d) Stimulation of B cell and T cell memory and their potential relevance for effective immune response to SARS-CoV-2.

SARS-CoV-2 is a family of enveloped, positive-sense, single-stranded, largest known RNA viruses in the Coronaviridae family. The genome size of SARS-CoV-2 ranged from 29.8 kb to more than 30 kb, and its genome structure followed the unique gene characteristics of known CoVs (Livingston and Bucher, 2020). CoVs are further categorised into four genera as the largest recognised RNA viruses: alpha, beta, gamma, and delta CoV (α-CoV, β-CoV, π-CoV), in the subfamily Coronavirinae. Both CoVs have a highly conserved genome structure with a single large 5 ′open reading frame (ORF) encoding a replicase of polyprotein followed by several additional ORFs encoding structural and accessory genes scattered throughout the genome's 3′ end (Yin and Wunderink, 2018). At 5′ among various genes of the CoV genome, the first gene occupies about two-third of the genome consisting of two large overlapping ORFs (ORF 1a and ORF 1b). There is a ribosomal frame-shifting signal at the junction of two ORFs. The viral genome translates into two large precursor polyproteins 1a and 1ab, which are processed by ORF 1a encoding for viral proteinases, papain-like proteinases (PLpro) and 3C-like proteinases (3CLpro), into 16 mature non-structural proteins (nsp1-nsp16) before entering the host cell. Out of these 16 nsps some performs essential function in viral replication (nsp3, nsp5, and nsp12) and transcription while others are RNA-processing enzymes (3′-5′ exoribonuclease, ribose 2-O Methyltransferase, Poly (U) specific endoribonuclease, cyclic nucleotide phosphodiesterase etc.). The enzymatic activities and functional domains of these nsps are preserved in different types of CoVs as they are essential during viral replication. The biological function of several nsps and their role in the life cycle of CoVs is still not characterized. On the other hand, 3′ of the genome encodes many structural virion proteins, including nucleocapsid (N), matrix (M) and envelope (E), as well as spike (S) proteins. Coronavirus S proteins are large membrane-linked glycoprotein's, responsible for binding host cells and membrane fusion to receptors. The S proteins are arranged in the S1 and S2 regions and are identical to these regions of both human immunodeficiency virus (HIV) and influenza proteins. The S1 domain of all described coronaviruses, including SARS-CoV-2, mediates the initial high-affinity interaction with their receptors. S-protein recruits human angiotensin-converting enzyme 2 (ACE2) as an input receptor and uses the TMPRSS2 (Transmembrane Protease Serine 2) cellular serine protease for S-priming protein (Wu and McGoogan, 2020; Dong et al., 2020). RRAR, a unique furin-like cleavage site (FCS) in the spike protein (S), which is absent in other lineage B βCoVs, such as SARS-CoV, is responsible for its high infectivity and transmissibility (Xia et al., 2020). Follis et al. (2006) examined the role of furin cleavage on the fusogenicity of the normally uncleaved SARS-CoVS glycoprotein. Introduction of a synthetic furin recognition sequence at R667 in the putative S1–S2 junctional region enabled efficient cleavage of the S glycoprotein to generate discrete S1 and S2 subunits and markedly increased the ability of the spike complex to mediate cell–cell fusion. The SARS-CoV-2/ACE2 interface was elucidated at the molecular level, and a key determinant of SARS-CoV2 transmissibility was found to be the efficiency of ACE2 usage as shown in Figure 1a.

## 3 Transmission and pathogenesis of SARS-CoV2

SARS-CoV2's origins are yet undisclosed, scientists are seeking to identify the animal host of COVID-19, but so far, no one is certain. However, through some reports it has been predicted that the outbreak might be related to the Seafood Market in Huanan South China. Some sources believe the likely host for the SARS-CoV-2 is bats, pangolins. Efforts are made to look for a host reservoir or intermediate carriers from which the virus is spread to humans. Initial reports identified two species of snakes, which might be a possible COVID-19 reservoir. However, no consistent evidence of CoV reservoirs has existed to date (Ouassou et al., 2020). COVID-19 genomic sequence analysis revealed 88% association with two bat-derived extreme CoV-like acute respiratory syndromes (SARS), suggesting that bats are the most likely source between COVID-19 and humans.

Many studies have suggested that human-to-human transmission of COVID-19 is a possible route for transmitting the infection. This is confirmed by cases that occurred within families and between individuals who did not attend the wet animal market in Wuhan. Several studies have indicated the most common cause of COVID-19 spread is asymptomatic people. In particular, it spreads among humans through coughing or sneezing from an infected person by respiratory droplets (Rothan and Byrareddy, 2020). The inhaled virus (SARS-CoV-2) possibly binds in the nasal cavity to the epithelial cells and begins replication. Its pathogenesis begins with the binding of the cellular receptor, commonly referred to as the angiotensin-converting enzyme 2 (ACE2) receptor expressed by the host cells and the virus spike proteins followed by their fusion (Wan et al., 2020; Jaimes et al., 2020). Importantly, the sequence of the COVID-19 spikes receptor-binding domain resembles that of previous SARS-CoVs (Wan et al., 2020). The virus binds to ACE2 through spike protein and allows COVID-19 to enter and infect cells. An enzyme called protease must be primed to the spike protein. SARS-CoV-2 uses a protease called TMPRSS2 similar to previous SARS-CoVs to complete their infection process. The genome of the virus is transcribed and translated after the virus enters the host cell and uncoats. Coronavirus genome replication and transcription occurs on cytoplasmic membranes as shown in Figure 1b and 1c and involves coordinated processes of both continuous and discontinuous RNA synthesis, regulated by the viral replicate, a huge protein complex encoded by the 20-kb gene. Up to 16 viral subunits and several cellular proteins make up the replicase complex. In addition to RNA-dependent polymerase, RNA helicase, and protease activity common to RNA viruses, coronavirus replicase was recently predicted to use a variety of RNA-dependent processing enzymes not (or rarely) found in other RNA viruses, including putative sequence-specific endoribonuclease, 3′ to 5′ exoribonuclease, 2′-O-ribose methyltransferase, ADP ribose 1′-phosphatase, and cyclic phosphodiesterase activities. The proteins are assembled at the cell membrane, and genomic RNA is incorporated as the mature particle forms by budding from the inner cell membranes (Mousavizadeh and Ghasemi, 2020).

## 4 Adaptive immune response against to COVID-19

Recent study by Suthar et al., demonstrated that the results coming from various studies on immune response to COVID-19 is so far unsurprising. The Immune system has numerous ways to detect and avoid the return of infectious pathogens. It selects B cells to produce antibodies that bind to the virus. It also extracts away a store of memory B cells that jumps into action if the virus attacks again. Beside, B cell another defensive mechanism was shown by T cells that patrol the body seeking out and destroying infected cells and unable the virus to replicate as shown in Figure 1d. Few current and pre-existing studies are highlighting that adaptive immune response could be one of the ways to provide defense against COVID-19. B-cells are as important as T-cells and are much more than just a final clean-up crew. They make important molecules called antibodies. These molecules trap specific invading viruses. Each B cell produces a single species of antibody, each with a unique antigen-binding site. When the memory B cell is activated by antigen (with the aid of a helper T cell), it proliferates and differentiates into an antibody-secreting effector cell.

### 4.1 Recent studies on SARS-CoV-2

Most recently, a retrospective analysis by Addetia et al., of SARS-CoV-2 outbreak on 71 fishing vessels that departed from Seattle, Washington in May 2020 furnishes first evidence that antibodies protect humans against re-infection. In this study, during pre-departure of the fishing vessel, serologically testing on 72 crew members was done and found that all were negative for SARS-CoV-2 out of 74, but an outbreak hit the vessel soon after it left the shore. Later, post-voyage testing showed that those members who were infected and had been tested positive before showed neutralizing antibodies against SARS-CoV-2 (Addetia et al., 2020). Antibody responses to SARS-CoV-2 can be detected in most infected individuals 10–15 days after the onset of COVID-19 symptoms. However, due to the recent emergence of SARS-CoV-2 in the human population, it is not known how long antibody responses will be maintained or whether they will provide protection from reinfection. Antibody responses to other human coronaviruses have been reported to wane over time. In particular, antibody responses targeting endemic human α- and β-coronaviruses can last for as little as 12 weeks18, whereas antibodies to SARS-CoV and MERS can be detected in some individuals 12–34 months after infection. Cross-sectional studies in SARS-CoV-2-infected individuals have so far reported lower mean neutralizing antibody titres for serum samples collected at later time points POS (23–52 d) (Seow et al., 2020).

The problem whether adaptive immune responses to SARS-CoV-2 are defensive or pathogenic is still extremely unclear, or whether both scenarios will arise depending on the nature, structure or severity of the adaptive immune response. Based on the available clinical data from SARS, MERS etc. this hypothesis ranges the full gamut. Other than this many other studies on adaptive immune response to SARS-CoV-2 has been published which includes the study by Grifoni et al., in the USA, reporting highest T cell reactivity against proteins of SARS-C0V-2 other than the spike protein. Little T cell reactivity was detected against spike protein and most of the T cell reactivity was associated with CD4+ T cells with smaller contribution by CD8+ cells (Grifoni et al., 2020). Similarly, the study by Weiskopf et al., in Netherlands’ observing CD4+ T cell reactivity against spike peptides in one of the ten unexposed subjects and against non-spike peptides in 2 out of 10 subjects. CD8+ T cell reactivity was observed in 1 out of 10 unexposed subjects (Weiskopf et al., 2020). Le Bert et al. in Singapore reporting T cell response to structural protein (nucleocapsid) and non-structural proteins (nsp7 and nsp13) in convalescence from COVID -19 patients (n = 24). In all of them demonstrated the presence of CD4+ and CD8+ T cells recognizing multiple region of nucleocapsid protein and showed that SARS-CoV-2 recovered patients (n = 23) still possess long lasting memory T cell reaction in 50% of the individuals with no history of COVID-19 (Bert et al., 2020). Meckiff, B.J. et al., in UK, reporting increased proportion of cytotoxic follicular cells and cytotoxic T helper cells (CD4+ CTLs) responding to COVID-19 and reducing the proportion of SARS-CoV-2 reactive regulatory T cells. CD4+ CTLs being highly enriched for the expression of transcripts encoding chemokines involves recruitment of myeloid cells and dendritic cells to the site of viral infection and clears the infection (Meckiff et al.*,* 2020). Braun et al., in Germany reported the presence of CD4+ T cell response against spike protein in 34% of COVID-19 healthy donors in seronegative individuals. According to them, the biological role of pre-existing SARS-CoV-2 S-cross-reactive CD4+ cells remains unclear for now but they might represent the key to understanding vast divergent manifestations of COVID-19. Their suspected high rate of asymptomatic infections in children and adults might assume that these S-reactive CD4+ T cells have protective role in COVID-19 (Braun et al., 2020). A study at single center, Wuhan enrolling a cohort of 452 patients with severe COVID-19 displayed a significantly lower number of total T cells, both helper T cells and regulatory T cells. In particular, a decrease in regulatory T cells has been observed among helper T cells, with a more pronounced decrease depending on the severity of the cases, and in memory T cells, while the percentage of naive T cells has been found to increase. Naive and memory T cells are essential components of the immune system, whose equilibrium is crucial to sustaining a highly efficient defensive response. Naive T cells allow a large and tightly coordinated release of cytokines to defend against new and previously unrecognized infection, while memory T cells mediate antigen-specific immune response. A dysregulation in their balance, which favors naive T cells activity compared to regulatory T cells, may contribute greatly to hyper-inflammation. On the other hand, a reduction in memory T cells may be suggested in COVID-19 relapse, as many recurrences have been reported in recovered COVID-19 cases (Prompetchara et al., 2020).

### 4.2 Pre-existing studies on SARS and MERS

Many pre-existing studies published in the past on SARS and MERS adaptive immune response to SARS-CoV-2 may be fruitful to provide the evidence to fight against SARS-CoV-2. The study by Li et al., in 2008 reporting that CD8+ T cell responses were more frequent with greater magnitude than CD4+ T cell responses. In addition, virus-specific T cells from severe group appeared to be a core memory phenotype with a significantly higher frequency of polyfunctional CD4 + T cells (IFNÿ, TNFα, and IL-2) and CD8 + T cells (TNFα, and degranulated state) compared to the mild-moderate cells. Strong T-cell responses were significantly associated with a higher neutralizing antibody while more Th2-cytokines (IL-4, IL-5, IL-10) in the fatal group (Sallusto et al., 2010; Li et al., 2008). Shin et al., in 2019 reported that early rise of CD8 + T cells in MERS-CoV infection correlates with disease severity and that dominant Th1 type helper T cells are observed at the convalescent period (Shin et al., 2019). Zhao et al., in 2016 reported that in an animal model, airway memory CD4+ T cells specific for conserved epitope are protective against lethal challenge and can cross react with SARS-CoV and MERSCoV (Zhao et al., 2016).

From all these existing and pre-existing studies, it has been speculated that substantial T cell reactivity exists in many unexposed persons. SARS-CoV-2-specific T cells characterization in patients with COVID-19 have just started. It is reported that T cell reactivity was highest in against the SARS-CoV-2 spike protein, which was homologous to COVs (Bert et al., 2020). T cell immunity pre-existing to SARS-CoV-2 could be relevant as it might influence the COVID-19 severity. It is possible that the persons with high levels of pre-existing memory CD4+ T cells could recognize SARS-CoV-2 and could mount the faster response upon exposure to SARS-CoV-2 and thereby limiting COVID-19 severity. Various studies in which pre-existing immunity is measured could possibly address the role of pre-existing T cells memory against COVID-19. Anti-S IgG antibodies positively correlate with COVID-19 severity, along with the circulating levels of monocytes, neutrophils and eosinophils, but independent of circulating T cells or viral load. Several studies reported an overall increase in both IgM and IgG anti-SARS-CoV-2 spike (anti-S IgG), as well as with the presence of neutralizing IgG and IgA antibodies in COVID-19 patients (Barnes et al., 2020; Robbiani et al., 2020).

Previous evidence also suggests that the response of type Th1 is a key to effective control of SARS-CoV and MERSCoV, and possibly true of SARS-CoV-2. Since the majority of epitopes found for both viruses are focused on the viral structural proteins, this will be useful in mapping certain epitopes associated with SARS-CoV/MERS-CoV with SARS-CoV-2 ones. If one can distinguish overlapping epitopes among the three viruses, use of convalescent serum from recovered SARS or MERS patients would be advantageous for application in passive immunization. For T cell epitopes, this will aid in future to design a cross-reactive vaccine that will protect against all three humanCoVs (Catanzaro et al., 2020).

## 5 From immune response to vaccines against COVID-19

**Figure 2:**
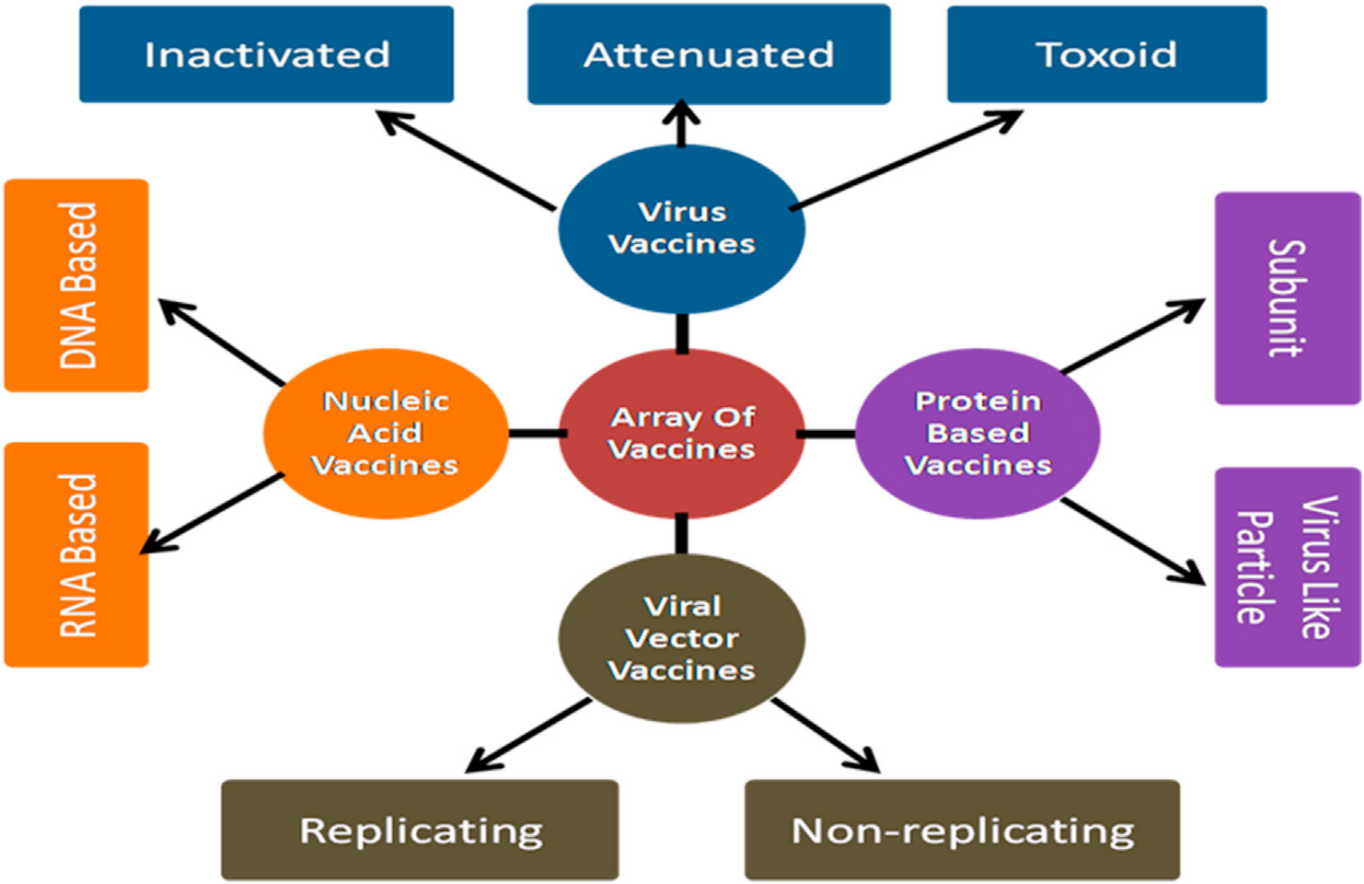
Different approaches for the development of vaccine for COVID-19.

Many new human pathogens have arisen in the last 20 years and, worryingly, pathogens that were previously suspected of being under control, such as diphtheria, influenza, and polio, has resurfaced. Vaccines are considered one of the most cost efficient strategies to combat infectious diseases. Efficient and effective vaccines capable of undertaking mass development include the possibility of eradication of certain diseases (Lauer et al., 2017). Suitable vaccines currently being developed and tested to deter SARS-CoV-2 infection include recombinant virus subunits, viral vector-based vaccines, and non-replicating virus particles, etc. On 11 August, COVID-19 vaccine **Sputnik V** developed by Gamaleya research institute in Mascow has been approved by Ministry of Health of Russian federation but as several considerable concerns about the efficacy and safety of vaccine has been raised and the vaccine yet has not been able to enter into PhaseIII of clinical trial. Currently 34 candidate vaccines are in clinical evaluation by 3 September (World Health Organization). Different approaches for the development of vaccines for COVID-19 shown in Figure 2 given below.

### 5.1 Virus based vaccines

Virus vaccines must be immunogenic, reasonably durable, healthy and effective to produce long-lasting immunity. To satisfy these criteria, studies of the vaccines need to have a thorough understanding of:

• The defensive functions of immune responses mediated by antivirals B and T-cells.
• The complexity and plasticity of major viral antigens
• Molecular biology and pathogenesis.

There are several types of vaccines, including full-inactivated viruses and live-attenuated virus vaccines, with different benefits and drawbacks in each. Although non-living virus vaccines have strong benefits of being healthy and durable, they may cause side effects and may be less effective relative to live-attenuated virus vaccines (Enjuanes et al., 2016). As several current vaccines such as the vaccine against measles, mumps and rubella (MMR) and the varicella (chickenpox) vaccine are made in this way. Toxoid vaccines may also be engineered to keep us immune from SARS-CoV-2 infection since they contain a bacteria or virus-created toxin or chemical. Currently five inactivated virus vaccines are in Phase II/III of clinical evaluation against COVID-19. **Sinovac Biotech- (NCT0044565595, PhaseIII), Beijing Institute of biological products (ChiCTR2000034780, PhaseIII), Institute of Medical Biology, Chinese Academy of Medical Sciences (NCT04470609, PhaseII), Research Institute for Biological Safety Problems, Rep of Kazakhstan (NCT 04530357, PhaseII), Bharat Biotech (NCT04471519, PhaseII)**. Together with this Codagenix in Farmingdale, New York, is collaborating with the Serum Institute of India to attenuate SARS-CoV-2 by altering its genetic code to make viral proteins less functional (Sarma et al.*,* 2020).

### 5.2 Nucleic acid vaccine

This has been a decade since researchers learned that the injection of 'naked' plasmid DNA and RNA without any related lipids, proteins or carbohydrates, could induce an immune response, they are usually referred as nucleic acid vaccines (Restifo et al., 2000). Currently, eight nucleic acids (DNA/RNA) are in Phase II/III of clinical evaluation. Previous Moderna research with patent application WO2017070626 on mice vaccinated with the full-length mRNA encoding protein S developed high neutralizing antibody titer in mice. Thus, in response to this Moderna announced that it has released the first batch of mRNA-1273 against SARS-CoV-2 for human use and is currently in clinical trial phase III. Moderna states that mRNA-1273 is an mRNA vaccine targeting a perfusion-stabilized version of the S-protein associated with SARS-CoV-2, selected by Moderna in collaboration with researchers at the National Research Center for Allergy and Infectious Diseases (NIAID). The Coalition for Outbreak Preparedness Innovations has funded manufacture of this package. Patent application WO2018115527 describes vaccines consisting of mRNA encoding at least one MERS coronavirus antigen, preferably an S protein or a S protein fragment (S1), an envelope protein (E), a membrane protein (M), or a nucleocapsid protein (N), all of which were successful in inducing antigen-specific immune response. It has been shown that intradermal administration into mice of a lipid nanoparticle (LNP)-encapsulated mRNA mixture encoding MERS-CoV S proteins contributes to in vivo translation and induction of humoral immune responses (Liu et al., 2020, Liu et al., 2020).

Besides Moderna, **BioNTech/Fosun Pharma/Pfizer (NCT04368728, PhaseIII), Curevac (NCT04515147, PhaseII), Inovio Pharmaceuticals/International Vaccine Institute (NCT044447781, NCT0433640, PhaseII), Osaka University/AnGes/Takara Bio (NCT04463472, NCT04527081/PhaseII), Cadila Healthcare Limited (CTRI/2020/07/026352, PhaseII), Genexine Consortium (NCT04445389, PhaseII), Arcturus/Duke-NUS(NCT04480957, PhaseII)** are working are working for the development of nucleic acid vaccine against COVID-19.

### 5.3 Protein based vaccine

Many vaccines currently available for human use are either virus-based or protein-based vaccines. Protein based vaccines consist of protein isolated from cells infected with viruses or bacteria, recombinant protein of virus-like particles (VLPs). VLP is composed of structural proteins essential for the development of virus particles but lacks viral genome and non-structural proteins. This vaccine needs an adjuvant to elicit a robust immune response to viral infection (Van Riel and Wit, 2020). Currently, one protein based vaccine which is Plant-derived VLP adjuvanted with GSK or Dynavax adjs. by **Medicago Inc. (NCT04450004, Phase I)** is in clinical evaluation while many others are in pre-clinical evaluation (PiCoVacc in preclinical phase).

### 5.4 Viral vector vaccines

It is understood that the viral vectors are potential methods for gene therapy and vaccines. Their use is based upon viruses' ability to infect cells. They have numerous advantages over others, such as transduction of high-efficiency genes, highly selective transfer of genes to target cells and activation of strong immune responses and increased cell immunity (Ura et al., 2014). The use of replicating or non-replicating viruses as a medium to produce an antigen/transgene vaccine is difficult. The big challenge to note is the possible activation of vector-specific immunity, which consequently impairs the ability of viral vectors to respond properly to the antigen/transgene. However, this issue can be minimized by following effective immunization methods, such as the use of viruses that do not replicate in humans or the use of specific viral serotypes for main and boosting immunizations (Choi and Chang, 2013). Owing to years of research adenoviruses has become one of the most commonly used vectors for vaccine production with its ability to target a diverse variety of hosts and cause elevated rates of transgene expression without the need for viral genes to be incorporated into the host genome. Similarly, Poxvirus, Alphaviruses is also used (Choi and Chang, 2013). Currently, three non-replicating viral vector vaccines are in clinical evaluation against COVID-19 by **University of Oxford/AstraZeneca (NCT04516746), CanSino Biological Inc./Beijing Institute of Biotechnology (NCT04526990, PhaseIII), Gamaleya Research Institute (NCT04530396, PhaseIII)**.

## 6 Repurposing drugs for COVID-19

No medications or vaccines are currently available for treating COVID-19. Studies are also focusing on repurposing current drug methods. Researchers working in this area have suggested the use of certain known broad-spectrum antiviral drugs such as nucleoside analogues, neuraminidase inhibitors and protease inhibitors as a possible path to treatment. In addition, important drug targets for the COVID-19 therapy are RNA-dependent polymerase and angiotensin-converting enzyme 2 (ACE2). Several antiviral medications such as Favinapir, Ritonavir, Oseltamivir, Lopinavir, Ganciclovir, Remdesivir, and Low Molecular Weight Heparin (LMWH) have clinically studied for infection with COVID-19 (Shah et al., 2020). Antiviral treatment helps to decrease symptoms and infectivity, and to reduce the duration of the disease. Such medicines function by stopping the cycle of viral replication at various stages. Most commercially available antiviral drugs are used to treat HIV infections, herpes viruses, hepatitis B and C viruses and influenza A and B viruses. Since viruses are attaching intracellular parasites, drug targets that interfere with viral replication without also harming the host cells are hard to identify. One of the studies conducted on 19 antiviral drugs demonstrates that not all the antiviral drugs have the direct antiviral effect on the COVID-19 (Ex-velpatasvir, ledipasivir, litonavir, lopinavir favilavir and sofosbuvir) but some have strong antiviral effect (Ex-Remdesivir, Chloroquine) (Liu et al., 2020, Liu et al., 2020).

Latest analysis of low molecular weight heparin (LMWH) antiviral drugs shows it enhances the coagulation dysfunction of COVID-19 patients by reducing and increasing the percentage of lymphocytes. By this study, Shi et al. indicated that LMWH could be used to treat COVID-19 as a potential therapeutic drug (Shi et al., 2020). Few Current updates on Antiviral Drugs for COVID -19 treatments are listed in Table 2.

**Table: 2:** Current updates on Antiviral Drugs for COVID -19 treatments.

Candidate Antiviral Drug	Type	Current Status
AT527 (Atea Pharmaceuticals)	Nucleoside analouge Highly selective, oral direct acting antiviral drug	The FDA cleared its Investigational New Drug Application (IND) for treating adults with mild COVID-19 disease on 20 May 2020, with one or more risk factors for poor outcome. Currently recruiting patients for Phase II clinical trial (NCT04396106) as of July 31, 2020. (https://clinicaltrials.gov/ct2/show/NCT04396106).
SLV213 (Selva Therapeutics)	Cysteine proteases such as cathepsin L, preventing the activation of viral spike protein and blocking viral entry into cells.	By July 21, 2020 after completing financing from private investors, the company said that it will proceed to be used towards rapidly advancing SLV123 into clinical trials as a leading drug candidate for treatment of COVID-19. (https://selvarx.com/selva/).
PAC-MAN (Stanford University)	CRISPR-based viral inhibition strategy.	Stanford University researchers have developed and screened CRISPER RNAs (crRNAs) targeting preserved viral regions and identified functional crRNAs targeting the virus - an approach that has been shown to effectively reduce the load of H1N1inflenza A virus in respiratory epithel cells. The study on this has been published in *Cell* detailing how Cas13d PAC-MAN effectively degraded RNA from SARS-COV-2 sequences and live influenza A virus in human lung epithelial cells. https://bioengineering.stanford.edu/news/scientists-aim-gene-targeting-breakthrough-against-covid-19
Ranpirnase (Orgenesis and Leidos)	Anti-viral broad-spectrum agent that catalyses RNA degradation and mediates multiple essential biological activities including control of cell proliferation differentiation, maturation and cell death.	The company has agreed on June 20, 2020 to develop its severe COVID-19 candidate drug through collaboration with Leidos ehose value is not disclosed yet but the company has submitted to FDA a pre-IND meeting request to fast track Ranpirnase for the treatment of COVID-19. https://investors.leidos.com/news-and-events/news-releases/press-release-details/2020/Orgenesis-and-Leidos-Collaborate-on-Potential-Treatment-for-COVID-19-/default.aspx
HTCC (N-(2-hydroxypropyl)-3-trimethlyammonium 47 chitosan chloride) (Jagiellonian and Nanjing University)	Antiviral compound conceived to suppress 48 currently circulating CoVs.	HTCC is a highly effective polymeric inhibitor to SARS-Covs-2. The study describing the antiviral activity of the HTCC as a potential inhibitor of 48 highly pathogenic CoVs based on its inhibition of viral replication in Vero cell has been published on March 21, 2020 from Nanjing university (Milewska et al., 2020). HTCC is a promising drug candidate that should be further studied, as it provides a ready-to-use solution for SARS-CoV-2 and future emerging CoVs, they said.
Antiviral drug candidates for COVID-19 (Anixa Biosciences and OntoChem)	The drug mainly targets two specific proteins of CoV: The protease, M^Pro^and an endonuclease shown to play a role in breaking up the RNA of SARS-CoV-2.	On July 6, 2020 the initial insilico screening of the drug has been completed and it has been identifies as an additional specific compound as well as multiple analog that could function as inhibitors of the main protease (M^pro^) of the COVID-19. In the study in June the company has said that they have identified four compounds that can disrupt the function of a viral enzyme endoribonuclease (Nsp-15). https://ontochem.com/2020/12/21/new-covid-19-therapy-drug-candidates-together-with-anixa-biosciences-inc/
OYA1 (OyaGen)	It is an antiviral drug with a wide spectrum. It shows activity against SARS-CoV-2 and MERS-CoV in lab dependent assay. It also shows activity as a dual target specific antiviral activity against Ebola virus.	On March 11, 2020 OyaGen, following unpublished positive results from collaborative research with NIAID at Fort Detrick, MD, announced for further testing of OYA1 for COVID-19. OyaGen suggests high dose-dependent antiviral activity of OYA1 against live SARS-CoV-2 based on its cell culture infectivity analysis. http://www.oyageninc.com/wordpress/
Arbidol (umifenovir) (Pharmastandard)	Membrane fusion inhibitor developed as influenza therapy.	Arbidol is currently in Phase IV of clinical trails as monotherapy and in combination that includes ASC09, lopinavir, ritonavir, cammycin and Bromhexine Hydrochloride. https://clinicaltrials.gov/ct2/show/NCT04350684
Brilacidin (Innovation Pharmaceuticals)	Formulation of antiviral small molecule drug, and COVID-19 vaccine containing mimetic defensin. The drug is in phase II development in head and neck cancer with oral muscositis.	On May 26, 2020 the company has received the data from an “unspecified leading public health research institute” showing the drug had inhibited SARS-CoV in a human cell line. The drug in comparision to vechical control showed an inhibitory effect on SARS-CoV-2 in a dose dependent manner with an average of 29% inhibition at 0.1ug/ml (lowest concentration) to an 85% inhibition at 100ug/ml (highest concentration). http://www.ipharminc.com/press-release/2020/11/30/innovation-pharmaceuticals-covid-19-clinical-trial-to-support-additional-development-of-brilacidin-as-a-pan-coronavirus-therapeutic
DAS181 (Ansun Biopharma)	Recombinant sialidase for the treatment of severe COVID-19 patient with strong antiviral properties	The drug is currently under Phase III clinical trial (NCT03808922) in hospitalized immunocompromised patients with para-influenza virus infection. Around 280 patients are in Phase IIb STOP Flu trial in China (NCT04298060) assessing the drug in severe influenza SAR-RV infection and COVID-19. https://www.ansunbiopharma.com/
Antiviral therapy based on company's novel nanomedicines platform. (Nano Viricidies)	Broad-spectrum virus-binding ligand: “It is like a ‘Venus-Fly-Trap’ for the virus,”	On May 12, 2020 the company has developed two drug candidates showing very high antiviral effectiveness in cell culture studies against multiple CoVs. The unrelated candidates when used against two unrelated CoVs that causes human disease- (hCoV-NL63)which uses the same receptor as SARS-CoV-2) was a good surrogate model for therapeutics development against SARS-CoV-2. The other CoV was hCoV-229E, causing seasonal common cold in humans. Nano Viricides said that it is planning to study its effectiveness against SARS-CoV-2 and will perform animal studies for its toxicology and safety.
Antiviral antibody treatment targeting COVID-19 (Celltrion Healthcare)	Monoclonal antibody	On July 17, 2020, in partnership with Chungnam National University Hospital in the third quarter, the Celltrion group launched Phase I trial to test a possible antiviral antibody treatment for CoVID-19 in 32 healthy volunteers, following positive preclinical results and subsequent approval of the company's application for IND by the Korean Ministry of Food and Drug Safety.. The antibody treatment will also be studied for use as a preventive measure to engage people internationally in close contact with COVID-19 patients. https://www.biospace.com/article/releases/nanoviricides-s-broad-spectrum-antiviral-drug-candidate-for-the-treatment-of-covid-19-infections-was-well-tolerated-in-glp-and-non-glp-animal-safety-studies/
Broad-spectrum antiviral compounds (Cocrystal and Kansas State University Research Foundation)	Protease inhibitors	Cocrystal extended its licencing agreement with the Kansas State University Research Foundation on April 22, 2020 to include access to additional preclinical leads and to further develop patented broad-spectrum antiviral compounds for COVID-19 therapy. .The compounds showed broad-spectrum activity against SARS-CoV-2, SARS-CoV, and MERS-CoV, as well as data on in vivo efficacy in an animal model MERS-CoV that was recently used for in vivo study of remdesivir. https://www.k-state.edu/media/newsreleases/2020-02/cocrystal_license22820.html
Ivermectin (Monash Biomedicine Discovery Institute (BDI) and Peter Doherty Institute of Infection)	Anti-parasitic drug approved by the FDA for Strongyloidiasis of the intestinal tract, and onchocerciasis (river blindness).	On April 3, 2020, the BDI and Doherty Institute research showed that Ivermectin effectively stopped the development of the SARS-CoV-2 virus in cell culture and within 48 h, significantly reducing the viral RNA at 24 h (Caly, 2020). The in vitro research will be followed up within 48 h with human clinical use and will minimize trials, the institutions said.
Chloroquine phosphate (Bayer as Resochin)	Bayer discovered resochin and introduced malaria medication into clinical practice in 1947. It is chloroquine phosphate salt, a quinoline compound with antimalarial and anti-inflammatory properties. .	On June 15, 2020, the FDA revoked the authorization for emergency use of Chloroqine phosphate and hydrochloroquine sulphate donated to Strategic National Stockpile for use in treating some hospitalized COVID-19 patients. https://clinicaltrials.gov/ct2/show/NCT04344951
Danoprevir (Ascletis Pharma)	Oral hepatitis C virus protease inhibitor	Ascletis trumpeted promising findings from a Chinese clinical trial on March 24,2020, the first such analysis of Danoprevir in COVID-19 patients, testing Danoprevir as opposed to ritonavir (NCT04291729). The company reported that after 4–12 days of Danoprevir treatment in conjunction with ritonavir, all 11 patients with moderate COVID-19 had discharged two treatment-naive, nine patients experienced were discharged from the hospital. The first negative RT-PCR test occurred at an average of 2 days, with occurrences varying from 1 to 8 days and CT scans absorption occurred at an average of 3 days, varying from 2 to 4 days. The trial was held at Nanchang The Ninth Hospital. Ascletis declared on 26 February 2020 the discharge of the first three patients of the trial.https://clinicaltrials.gov/ct2/show/NCT04291729
Kaletra (lopinavir-ritonavir) (AbbVie-Kaletra)	In conjunction with other antiretroviral agents, the HIV-1 protease inhibitor was indicated for treating HIV-1 infection in adults and children 14 days of age and older.	University of Oxford researchers conducting the the Randomised Evaluation of COVID-19. The preliminary results on Lopinavir/Ritonavir yields no significant mortality benefits in hospitalized COVID-19 patients was relapsed on June 30, 2020. “Lopinavir-ritonavir is not an effective treatment”, declared Oxford Prof. Peter Horby, MD, PhD, the chief investigator for the trial, which enrolled over 11,800 patients from 176 NHS hospitals in the U.K. Vanden Eynde, Jean J. 2020. "COVID-19: An Update about the Discovery Clinical Trial" *Pharmaceuticals* 13, no. 5: 98. https://doi.org/10.3390/ph13050098
Favipiravir (AppiliThrapeutics)	Anti-viral broad-spectrum agent engineered to selectively and potently inhibit RNA-dependent RNA polymers from RNA viruses.	On August 10, 2020, Appili Therapeutics was granted FDA clearance to extend its Phase II clinical trial (NCT04448119) into the U.S. to evaluate the safety and efficacy of favipiravir tablets in long-term care facilities in managing outbreaks following exposure to COVID-19. Appili said it plans to enrol up to 760 trial participants throughout the U.S. as well as in Canada. On May 21 Health Canada issued regulatory clearance for the Phase II study designed to assess the favipiravir of Fujifilm Toyama Chemical as a preventive measure against COVID-19 outbreaks. https://clinicaltrials.gov/ct2/show/NCT04448119
Combination of Plaquenil (hydroxychloroquine sulfate) and Zithromax (azithromycin); Chloroquine phosphate; Chloroquine hydrochloride; Zithromax; Zmax Sanofi, Pfizer, and various manufacturers	Plaquenil and the chloroquine treatments are antimalarial drugs; plaquanil has also been approved by the FDA for lupus erythematosus and rheumatoid arthritis. Chloroquine, for extraintestinal amebiasis. Zithromax and Zmax are antibacterials indicated for adults with acute bacterial sinusitis, and community-acquired pneumonia. Zithromax is also indicated for acute bacterial exacerbations of chronic obstructive pulmonary disease.	On August 14, 2020 Taiwan Liposome (TLC) submitted an IND to the Taiwan FDA for TLC19 Hydroxychloroquine Liposome Inhalation Suspension for the treatment of COVID-19. TLC19 uses TLC's existing proprietary liposome technology to encapsulate ~1/100 of the oral hydroxychloroquine (HCQ) dose into an inhalable formulation for direct deposit into the airways and lungs. TLC reasons that TLC19 may have an antiviral effect with a miniscule dosage relative to oral HCQ thus minimising blood and heart exposure, giving TLC19 the ability to treat COVID-19 without many of the side effects found in some trials (Tai et al., 2020).

## 7 Immunomodulators

The treatment possibilities for extreme COVID-19 remain minimal to date. A variety of antiviral medications, such as lopinavir/ritonavir, has demonstrated little benefit relative to routine treatment (Cao et al., 2020). A new treatment approach is likely to be in effect in addition to antiviral therapy alone, which is about to have a significant impact on health outcomes. Immunomodulatory treatment for controlling the "cytokine storm" can provide insights into COVID-19 treatment. The combined use of immunomodulatory agents with antiviral agents to minimize cytokine storm may also provide effective care for COVID-19 treatment (Zhao, 2020). Some of the study based approach to Immunomodulatory drugs treatment against COVID-19 as given below:

### 7.1 Tocilizumab

Between February 21 and March 24, 2020, an observational cohort study with severe COVID-19 pneumonia patients in Bologna and Reggio Emilia, Italy showed that if the medication administered intravenously or subcutaneously might minimise the risk of intrusive mechanical ventilation or death in patients with severe COVID-19 pneumonia (Guaraldi et al., 2020).

### 7.2 Infliximab (CT-P13)

The study on patients with rheumatic disease diagnosed with COVID-19, anti-TNF use was associated with decreased hospitalization chances (OR 0.40, 95 per cent CI 0.19 to 0.81). Similar to an Italian case study of an adult patient with serious ulcerative colitis and COVID-19 after 7 days on infliximab progressively recovered from the effects of the instinct and substantially improved overall well-being. In the cytokine storm associated with extreme COVID-19 infection, these emerging data demonstrate a critical role for anti-inflammatory medication. Due to its broad availability and well known safety profile, CT-P13 has been used for many years in the treatment of multiple inflammatory conditions making it a strong candidate for COVID-19 therapy (Feldmann et al., 2020).

### 7.3 Dupilumab

When the drug tested for patients with uncontrolled, moderate to severe asthma, is an IL4 and IL-13 antagonist, which has proven to reduce severe exacerbation rates and improved forced expiratory volume (FEVI). Hawill et al., by using transfer learning concluded that the timing of administration of the drug is critical and could be efficacious during the later stages of the infection so that cytokine storms could be mediated especially, in older patients (Hawill and Geraci, 2020).

### 7.4 Certolizumab

Based on the features of genetic homology and pathology of infected lung, Zhang et al. predicted that cytokine storm also prevails in COVID-19 patients. There was a marked increase in interleukins compared to SARS patients, which indicated some possible difference in CoV pathogenesis from SARS and MERS. Patients with hypoalbuminenia, lymphopenia, neutropenia and a reduced percentage of CD8 + cells also have possible repressed immune function in COVID-19. A clinical trial using Certolizumab pegol (a TNF blocker) along with antiviral therapies can have beneficial effects in patients with COVID-19 (Zhang et al., 2020).

### 7.5 Anakinra (ACZ885)

Several studies indicate that anakinra offers the greatest benefit in COVID-19 with the Cytokine Storm Syndrome feature when patients need high levels of supplements for <2 days before incubation, noting that "early aggressive treatment was probably crucial to improving patients' risk of mechanical ventilation without significant complications (Navarro-Millán et al., 2020; Cavalli et al., 2020; Julián et al., 2020).

### 7.6 Brodalumab, secukinumab and ixekizumab

All three of those drugs are available commercially. Secukinumab and ixekizumab are approved for psoriasis, psoriatic arthritis and spondylitis ankylosing while Brodalumab is approved for psoriasis treatment alone. All these are given with warning of an increased risk of infection. Therefore, the incidence of severe infection is unchanged or low over the short term; the use of these drugs in COVID-19 acute setting infection does not result in an increased risk of secondary infection (Pacha et al., 2020).

### 7.7 Immunosuppressant

Immunosuppressive agents along with immunomodulators minimise the amount of cytokines required to activate and differentiate the immune cells to clear the infection. Furthermore, inflammatory mediators can become hyperactive, leading to a 'cytokine storm’, which is the primary cause of death in severe diseases. Hence, for those affected by COVID-19, classic immunosuppressants may present the most serious risk (Price et al., 2020). Example:

### 7.8 Dexamethasone

It is glucocorticoid agonist having immunosuppressive, immune-inflammatory properties. The preliminary research on this drug found that dexamethasone decreased 28-day mortality in patients diagnosed with COVID-19 among those receiving randomization intrusive mechanical ventilation or oxygen but not among patients not receiving respiratory support (Horby et al., 2020).

### 7.9 Tacrolimus and cyclosporin

Massachusetts General Hospital's guidelines indicated that the dignified COVID-19 tacrolimus and cyclosporine doses should be decreased by 50 per cent in non-critically ill and liver transplanted patients and their plasma concentration should stay at 25–50 ng ml and 3–5 ng/ml, respectively (Mirjalili et al., 2020). As at this time, the immune system of these patients is already weak.

### 7.10 Tofacitinib

Tofacitinib is a Janus kinase (JAKs) 1 and 3 inhibitor that has partial selectivity to JAK 2. It suppresses pro-inflammatory signals that may be pathogenetically important for progression to more serious lung disease and ARDS in COVID-19 patients (https://clinicaltrials.gov/ct2/show/NCT04469114).

### 7.11 Rapamycin (sirolimus)

An immunosuppressive mTOR inhibitor drug that is designed to treat T cell (CD4+ and CD8+) dysfunction in patients with COVID-19. In extreme COVID-19 patients, cytokine storm may promote T-cell apoptosis, necrosis or pyroptosis that causes reduction in T-cell counts and is inversely associated with survival of the patient and in this case, rapamycin may promote autophagy and reserve senescence of T-cells (Omarjee et al., 2020).

Currently there is not any clear data for many of these immunomodulators/immunosuppressive drugs that explain the advantages or risks of stopping these drugs during the COVID-19 pandemic outbreak. Nonetheless, the mode of action of each drug, method/frequency of administration and pharmacokinetics/pharmocodynamics is important to consider.

## 8 Conclusion and future perspective

Considering the ongoing COVID-19 disease pandemic and raising the death risk scenario, the discovery of new drugs and the production of vaccines is a time-constrained problem. While much progress has been made on SARS-CoV-2, with clear basic requirements for their capacities during the life cycle of the CoV, as well as the structures behind their pathogenesis to their simplicity of transmission, there is a need for further analysis of CoV molecular biology, which can help to produce effective anti-coronaviral agent against COVID-19. Since Effective immune cells memory can persist for a long period resulting in accelerated immune responses and enhanced control of SARS CoV-2, generation of strong and sturdy T and B cell memory is a target of vaccines, including the numerous vaccines against SARS-CoV-2 that are currently in human trials.

## Declarations

### Author contribution statement

All authors listed have significantly contributed to the development and the writing of this article.

### Funding statement

Rohit Saluja was supported by the 10.13039/501100010803Department of Biotechnology, India for Ramalingaswami Re-entry Fellowship (BT/RLF/Re-entry/53/2013).

### Data availability statement

Data included in article/supplementary material/referenced in article.

### Declaration of interests statement

The authors declare no conflict of interest.

### Additional information

No additional information is available for this paper.
